# Exploring the research landscape of the past, present, and future of thyroid nodules

**DOI:** 10.3389/fmed.2022.831346

**Published:** 2023-01-12

**Authors:** Pei Chen, Chenzhe Feng, Leyi Huang, Haolin Chen, Yeqian Feng, Shi Chang

**Affiliations:** ^1^Department of General Surgery, Xiangya Hospital Central South University, Changsha, Hunan, China; ^2^Department of Mathematics, University of California, Davis, Davis, CA, United States; ^3^Department of Oncology, The Second Xiangya Hospital of Central South University, Changsha, Hunan, China; ^4^Clinical Research Center for Thyroid Disease in Hunan Province, Changsha, Hunan, China; ^5^Hunan Provincial Engineering Research Center for Thyroid and Related Diseases Treatment Technology, Changsha, Hunan, China; ^6^National Clinical Research Center for Geriatric Disorders, Xiangya Hospital, Changsha, Hunan, China

**Keywords:** thyroid nodules, machine learning, natural language processing, publication analysis, PubMed

## Abstract

**Introduction:**

The purpose of this study was to assess the landscape of thyroid nodules research during the last 22 years using machine learning and text analysis.

**Methods:**

In November 2021, we obtained from PubMed all works indexed under the Medical Subject Headings (MeSH) subject line “thyroid nodules.” The entire set of search results was retrieved in XML format, and metadata such as title, abstract, keywords, MeSH words, and year of publication were extracted for bibliometric evaluation from the original XML files. To increase the specificity of the investigation, the Latent Dirichlet allocation (LDA) topic modeling method was applied.

**Results:**

Our study included 5,770 research papers. By using frequency analysis of MeSH terms, research on thyroid nodules was divided into two categories: clinical and basic. The proportion of clinical research is nearing 89% and is dominated by the differential diagnosis of thyroid nodules. In contrast, the proportion of MeSH terms relating to basic research was just 11%, with DNA mutation analysis being the most common topic. Following this, LDA analysis revealed the thyroid nodule study had three clusters: Imaging Studies, Biopsy and Diagnosis, and Epidemiology and Screening of Thyroid Cancer. The result suggests that current thyroid nodule research appears to have focused on ultrasonography and histological diagnosis, which are tightly correlated. Molecular biomarker research has increased, therefore enhancing the diagnostic precision of thyroid nodules. However, inflammation, anxiety, and mental health disorders related to thyroid nodules have received little attention.

**Conclusion:**

Basic research on thyroid nodules has unmet research requirements. Future research could focus on developing strategies to more efficiently identify malignant nodules, exploring the mechanism of thyroid nodule development, and enhancing the quality of life of thyroid patients.

## 1. Introduction

Thyroid nodules are one of the most prevalent diseases diagnosed at physical examination, with approximately 5% of the population having palpable nodules (1) and up to 68% having ultrasound detectable lesion patterns (2). Over the past two decades, researchers have created validated assessment methods for thyroid nodules, Thyroid Imaging Reporting and Data System (TI-RADS) (3, 4) and Bethesda (5), based on ultrasound imaging features and cytological microscopy examination. Meanwhile, molecular features of thyroid nodules have been observed, such as the BRAF (V600E) mutation, the RET/PTC rearrangement, the RAS mutations, and etc. Up until now, thyroid nodules have been challenging to diagnose and treat because of the rapid increase in populations.

Bibliometrics is an interdisciplinary discipline that uses mathematical and statistical methodologies to conduct quantitative analyses of knowledge vectors. A bibliometric study is a type of research that aims to offer a quantitative overview of disciplinary fields. Bibliometrics has a remarkable edge on cluster analysis of calculating document and author quantities, and word frequencies, because of which it has been widely used to demonstrate the research status in variable areas. Through long time and frequent use of this methodology, bibliometrics has also raised new inquiries and pushed forward the development of different areas.

Natural language processing (NLP), a frequently used approach in artificial intelligence for investigating research papers (6), has been well applied to evaluate study profiles in cancer rehabilitation (7) and glioma (8). Unlike prior studies, we focus on the variations of topics in thyroid nodules rather than on the differences across locations and authors of scientific texts. Using text analysis and NLP methods, this study is set to explore the research status of the thyroid nodules and we hope this research can help to point out new changes in research directions, present limitations of current research, and seek to identify future topics worth further exploration.

## 2. Materials and methods

This study used the methods of previous studies (7, 8). We downloaded the whole dataset indexed under the Medical Subject Headings (MeSH) topic phrase “thyroid nodules” from PubMed for the years 2000 to the present. The entire record of the search results is accessible in XML format. Meanwhile, the title, abstract, keywords, year of publication, and MeSH terms in the paper were extracted from the XML data.

To effectively identify the themes of each publication, we used the traditional the Latent Dirichlet allocation (LDA) model approach for topic extraction. LDA is an unsupervised machine learning technique that can be applied to large-scale document collections or corpora to identify latent topic information (9). This method helps the interpretation each document as a word frequency vector, allowing textual data to be converted into numerical data that can be easily modeled. In the three-level structure of words, topics, and documents, each document represents a probability distribution composed of some topics, and each topic represents a probability distribution composed of many words. As a result, depending on the frequency of feature words in each document, the LDA algorithm calculates the frequency that an article examines a specific study topic.

In the algorithm of LDA, the number of topics is generally inconsistent for different categorization tasks of different topics (9). In this study, we used four approaches to estimate the best number of themes (Supplementary material), and we decided on 20 as the final number. Based on the analysis of the article abstracts, we defined the main topic of each article with the highest probability calculated by LDA. Following the naming of the subjects, we use the Louvain algorithm for cluster analysis to create a network based on topic similarity and identify common communities of linked topics based on their association (10).

The relevant Python and R language code can be found in the cited literature (7, 8). Descriptive statistics are reported as mean ± standard deviation. Excel and Gephi^1^ were used to create the network visualization in this article (11, 12). An institutional review board or an ethics committee was not required as this is a bibliometric analysis-type study.

## 3. Results

**FIGURE 1:**
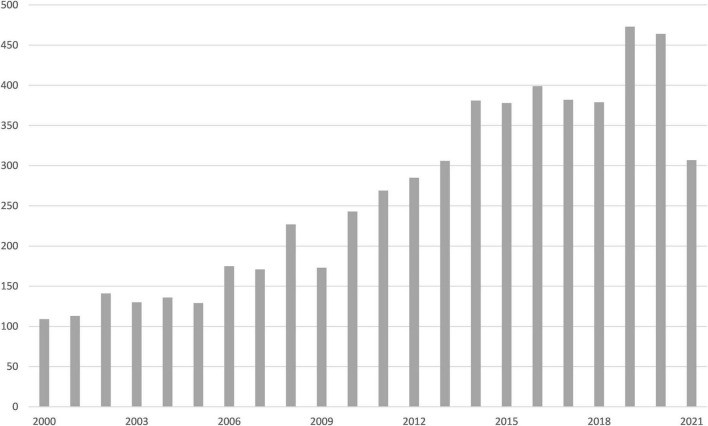
PubMed search results: articles per year.

This research included 5,289 journal articles, 119 clinical trials, 65 randomized controlled trials, 109 multicenter studies, 567 case reports, and 689 reviews (Figure 1). Between 2000 and 2009, there were 150 publications published annually on average. However, in the last 5 years, that number has increased to 401, indicating a growing interest in thyroid nodules.

### 3.1. MeSH analysis

**FIGURE 2:**
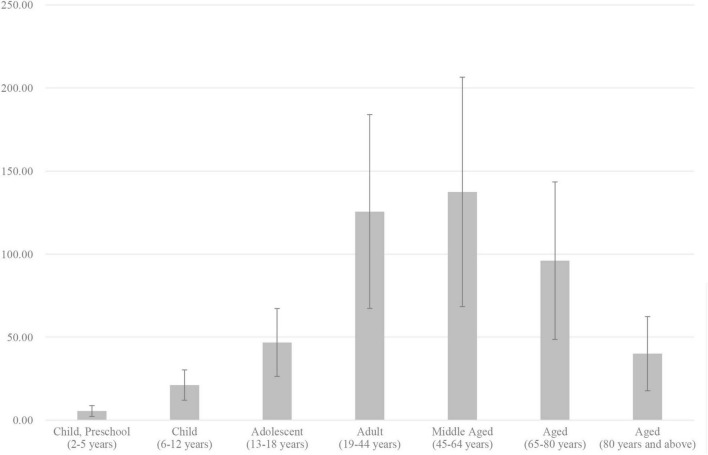
Annual output of literature, broken down by age group.

First of all, we excluded several uninformative words from the MeSH words analysis for further study, such as thyroid and thyroid nodules. Figure 2 depicts the percentage of publications connected to different age groups of the study population in the last 22 years. It is worth noting that the research target population is the adult and middle-aged population, followed by the elderly (65–80 years). The top 20 research themes for thyroid nodules are shown in Table 1. Currently the most common topics include Thyroid Neoplasms, Fine-Needle Biopsy, Ultrasonography, Retrospective Studies, and Differential Diagnosis.

**TABLE 1:** Overall ranking of research foci in the past 22 years.

Rank	MeSH headings	Record of occurrence in publications	% of total
1	Thyroid Neoplasms	2,896	50
2	Biopsy, Fine-Needle	2,190	38
3	Ultrasonography	1,627	28
4	Retrospective Studies	1,531	27
5	Diagnosis, Differential	1,133	20
6	Sensitivity and Specificity	1,035	18
7	Thyroidectomy	1,020	18
8	Carcinoma, Papillary	721	12
9	Predictive Value of Tests	526	9
10	Reproducibility of Results	497	9
11	Treatment Outcome	478	8
12	Adenocarcinoma, Follicular	470	8
13	Prospective Studies	446	8
14	Follow-Up Studies	410	7
15	Thyroid Cancer, Papillary	361	6
16	Carcinoma	360	6
17	Risk Factors	350	6
18	Biopsy, Needle	310	5
19	Biomarkers, Tumor	309	5
20	Prognosis	304	5

**FIGURE 3:**
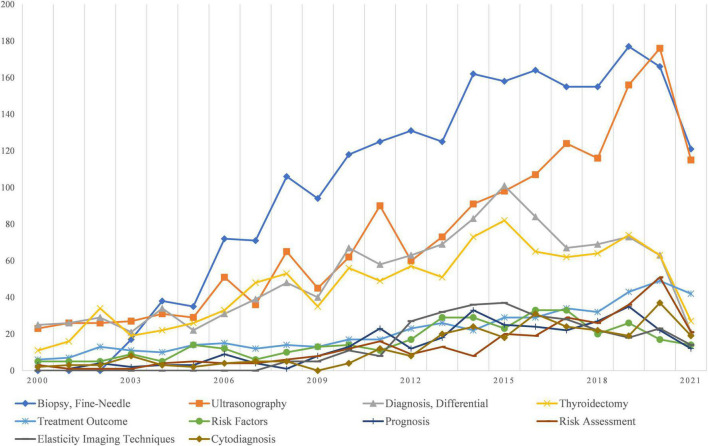
Research foci trends related to clinical research.

**FIGURE 4:**
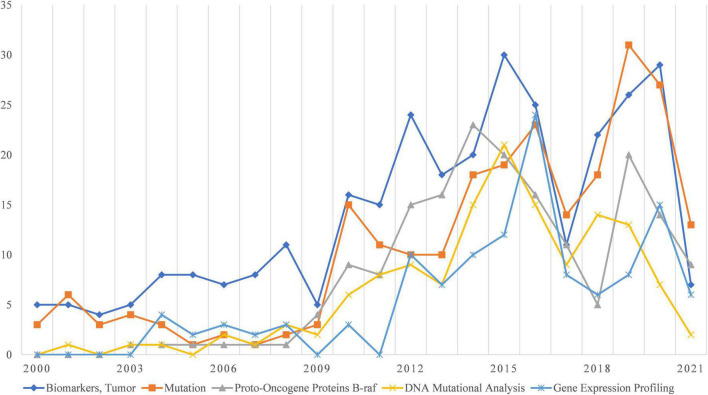
Research foci trends related to basic research.

According to the specific attributes, MeSH words with a total frequency >100 were analyzed from two perspectives: clinical research and basic research. Fine-Needle Biopsy and Ultrasonography were the most common clinically relevant MeSH terms (Figure 3). Differential Diagnosis was the third most common clinically relevant MeSH term. In addition, Treatment Outcome and Prognosis, as well as Risk Factors and Risk Assessment for thyroid nodules, have been the emphasis of the research. Ultrasound Elastography and Cytodiagnosis are also gaining popularity as time goes on. Related to basic research MeSH terms (Figure 4), the overall number is small, and there are only 5 terms that occurred in more than 2% of our dataset. Identification of biomarkers associated with cancers, especially by gene alterations such as DNA Mutational Analysis, is the most important subject in fundamental research. The study of Proto-Oncogene Proteins B-raf and Gene Expression Profiling is also a prominent issue.

**FIGURE 5:**
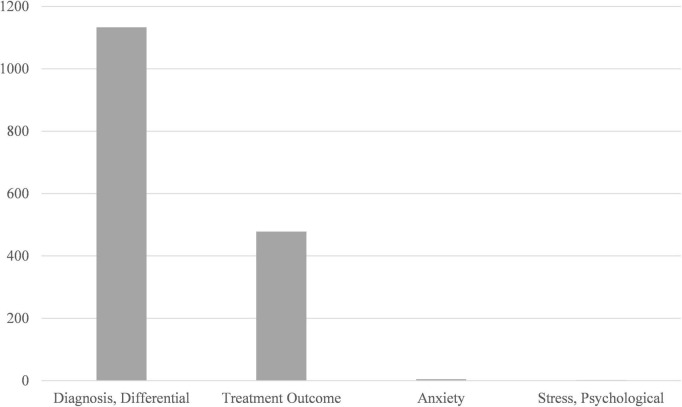
Comparison between the total amount of Differential Diagnosis, Treatment Outcome, Anxiety, and Psychological Stress.

**FIGURE 6:**
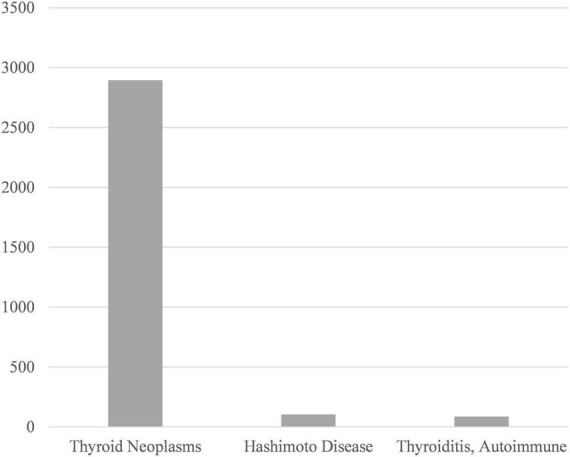
Comparison between the total amount of Thyroid Neoplasms. Hashimoto Disease, and Autoimmune Thyroiditis.

Our research also explored the process and outcome of thyroid nodule diagnosis, besides we observed some limitations of the existing studies. When compared to the most frequently mentioned academic terms, such as Differential Diagnosis and Treatment Outcome, Anxiety and Psychological Stress were less concerned (Figure 5). Likewise, Hashimoto Disease and Autoimmune Thyroiditis were found less frequently than Thyroid Neoplasms when process-related words were examined (Figure 6).

### 3.2. Latent Dirichlet allocation analysis

**FIGURE 7:**
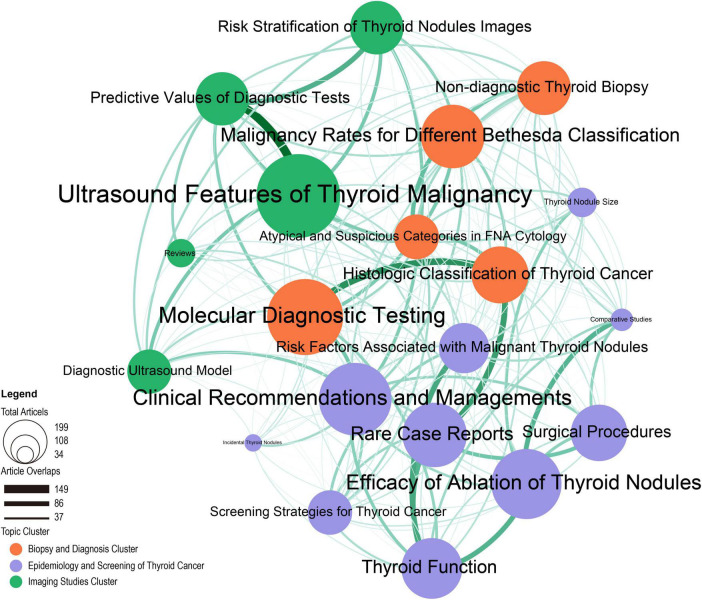
The Latent Dirichlet allocation (LDA) research topic cluster network: inter-and intra-relationships.

All of the themes were submitted for network analysis. The Louvain method was utilized to identify the thematic network clusters and obtain insights into the relationships between the prominent topics. Figure 7 shows three clusters in different colors: Imaging Studies, Biopsy and Diagnosis, and Epidemiology and Screening of Thyroid Cancer. The key theme in the Imaging Studies Cluster is Ultrasound Features of Thyroid Malignancy, which is closely related to Predictive Values of Diagnostic Tests and Risk Stratification of Thyroid Nodules Images. This indicates that the researcher is using nodule features defined by ultrasound examination-detectable features to create a diagnosis model. Meanwhile, in the Biopsy and Diagnosis Cluster, the most common theme is Molecular Diagnostic Testing, which is closely linked to Thyroid Cancer Histologic Classification. In addition, Rare Case Reports is another area of study that is linked to the Histologic Classification of Thyroid Cancer and Thyroid Function. This shows that in rare cases, research focuses on pathology and thyroid function changes.

## 4. Discussion

The prevalence of thyroid nodules has increased dramatically over the past two decades (13), from 1 to 5% in non-iodine-deficient regions (14) at the turn of the 21st century to 33–68% in randomized populations (2, 15). Since 2009, there has been a significant increase in the number of published papers, and the findings of LDA-related studies indicate that the majority of these changes are associated with thyroid nodule ultrasonography, biopsy, and diagnosis. This change is caused by a critical stage in the diagnosis and treatment of thyroid nodules.

It is well known that the most accurate and sensitive method for detecting thyroid nodules is diagnostic ultrasound imaging (16). This technique permitted the creation of numerous invasive procedures. In 2003, fine-needle aspiration biopsy (FNAB) became the “gold standard” for identifying thyroid nodule (16). Kim et al. (17) then reported on the use of transcatheter radiofrequency ablation for the treatment of thyroid nodules, further emphasizing the need of ultrasound technologies for thyroid nodule detection and treatment. However, because cytological examination is usually non-diagnostic due to inadequate specimens, this procedure reduces the sensitivity, specificity, and predictive potential of the diagnosis (18). In the 2006 version of the American Thyroid Association guidelines for the treatment of thyroid nodules and differentiated thyroid carcinoma, FNAB is often only recommended for nodules greater than 10–15 mm (19). This recommendation was amended in 2009, when Horvath et al. (20) proposed TI-RADS to identify ultrasonographic malignant characteristics of thyroid nodules, reducing the suspicious nodule diameter to 5 mm (3). Meanwhile, to boost the precision of preoperative diagnosis, the identification of molecular markers specific to thyroid cancer should be enhanced (21). These significant findings have prompted more in-depth research on the topic of thyroid nodule in terms of ultrasound imaging, diagnosis, and ultrasound-guided treatment. Also, one of the hottest topics in thyroid nodule research is Thyroidectomy, in which we noticed a huge interest in Rare Case Reports. Previous documents on the rare cases was shown in a strong correlation with Surgical Procedures and Histologic Classification. Such consisetent correlation provides solid evidence for researchers to identify the histological subgroups of thyroid cancer (22) and provide targeted therapeutic recommendations to restrict the scope of thyroid surgery (23).

With the thyroid nodule publications increasing, the more detailed MeSH analysis suggests that thyroid nodules arise at the same age as thyroid cancer, which is between 19 and 45 years of age (24). At the time of the initial diagnosis, 7–15% of the population is diagnosed with thyroid cancer. The majority of thyroid nodules are under active surveillance, with 1.1% turning into thyroid cancer (25). Patients with thyroid cancer usually can keep a long survival period due to the slow progression of the tumor. Thus in the long term, living quality, instead, attracts more attention since it will be threatened by fatigue and anxiety (26, 27). The current research on patients’ cancer phobia is relatively lagging. Further studies on the mental health of patients with thyroid nodules in various geographical and cultural contexts should be conducted in the future.

According to the MeSH subject and LDA model results of our research work, there are numerous treatment and diagnosis-related topics. However, basic research on thyroid nodules represents just a small portion of the medical literature on tumor markers such as BRAF (V600E) mutations, RET-PTC, and RAS (21), whose sensitivity and specificity are still insufficient (28). To guide more precise treatment, additional study into the mechanism of thyroid nodule formation, the creation of a more sensitive diagnostic model, and the establishment of a better assessment system are required.

Noteworthy as well is the high level of clinical diagnostic interest in thyroid nodules regarding thyroid tumors and the low level of interest in thyroid autoimmune diseases. In 2006, the census found that approximatively 10–15% of the population had Hashimoto’s thyroiditis (29). There is a growing body of evidence suggests that Hashimoto’s thyroiditis raises the probability of thyroid nodules and is a significant risk factor for thyroid cancer (30, 31), as well as increased incidence of mental problems such as depression and anxiety (32). This could be due to the fact that articles on Hashimoto’s thyroiditis did not include the keyword “thyroid nodules,” implying that thyroiditis warrants further investigation in the future.

This study has a number of limitations. For instance, bibliometric research can be conducted using databases other than PubMed, such as Web of Science, Scopus, and Embase. However, the advantage of PubMed is that it includes peer-reviewed studies of the highest quality and removes irrelevant, non-peer-reviewed papers (33). Future scholars will be able to provide a more thorough and extensive overview of the area than we have provided here if they have access to papers in other medical science databases. Moreover, we have also included a modest number of case reports and reviews. These might slightly increase the weight of duplicate studies during the analysis. In contrast, it also indicates that researchers are interested in the topic of thyroid nodules. Furthermore, due to the small number of basic research included, this report focuses on the number of publications without considering the Journal Citation Indicator and Impact Factor, which may diminish the contribution of high-quality articles to the research issues. Additionally, some newly released papers had not yet been indexed by MeSH words, so they may not have been included in our analysis. These are some of the most typical flaws in published research (7, 34). Lastly, in this work, artificial intelligence was employed to construct the LDA themes and their relationships, yielding a machine-driven understanding. A more comprehensive analysis of these themes could result in more accurate interpretations and implications, so enabling medical experts to give more effective therapies.

## Data availability statement

The raw data supporting the conclusions of this article will be made available by the authors, without undue reservation.

## Author contributions

YF and SC: conceptualization, writing—review and editing, and supervision. CF and PC: methodology, investigation, and writing—original draft preparation. CF, PC, LH, and HC: formal analysis. All authors contributed to the article and approved the submitted version.

## Footnotes

1
https://gephi.org/
