# Supplementary material for: Exploring the research landscape of the past, present, and future of thyroid nodules
Source: Front Med (Lausanne). 2023 Jan 12;9:831346. doi: 10.3389/fmed.2022.831346 (PMC9877524; doi:10.3389/fmed.2022.831346)
Supplement: Supplementary file 1 [file Data_Sheet_1.pdf]

Supplementary Material

The number of topics is usually determined by the purpose of classifying tasks. The topic classification algorism to classify the publications is unsupervised learning, so there is no way to perform the accurate correction and compare the accuracy of different topic numbers modeling. However, there are indeed some methods that explored the best topic number. Here we used four different approaches to identify the number of the topics[1-5].

From this figure, we can see those different methods identify different numbers of topics, and there is no consistent best number of topics. Based on our own experience, we set the number to 20.

Supplementary Figure 1

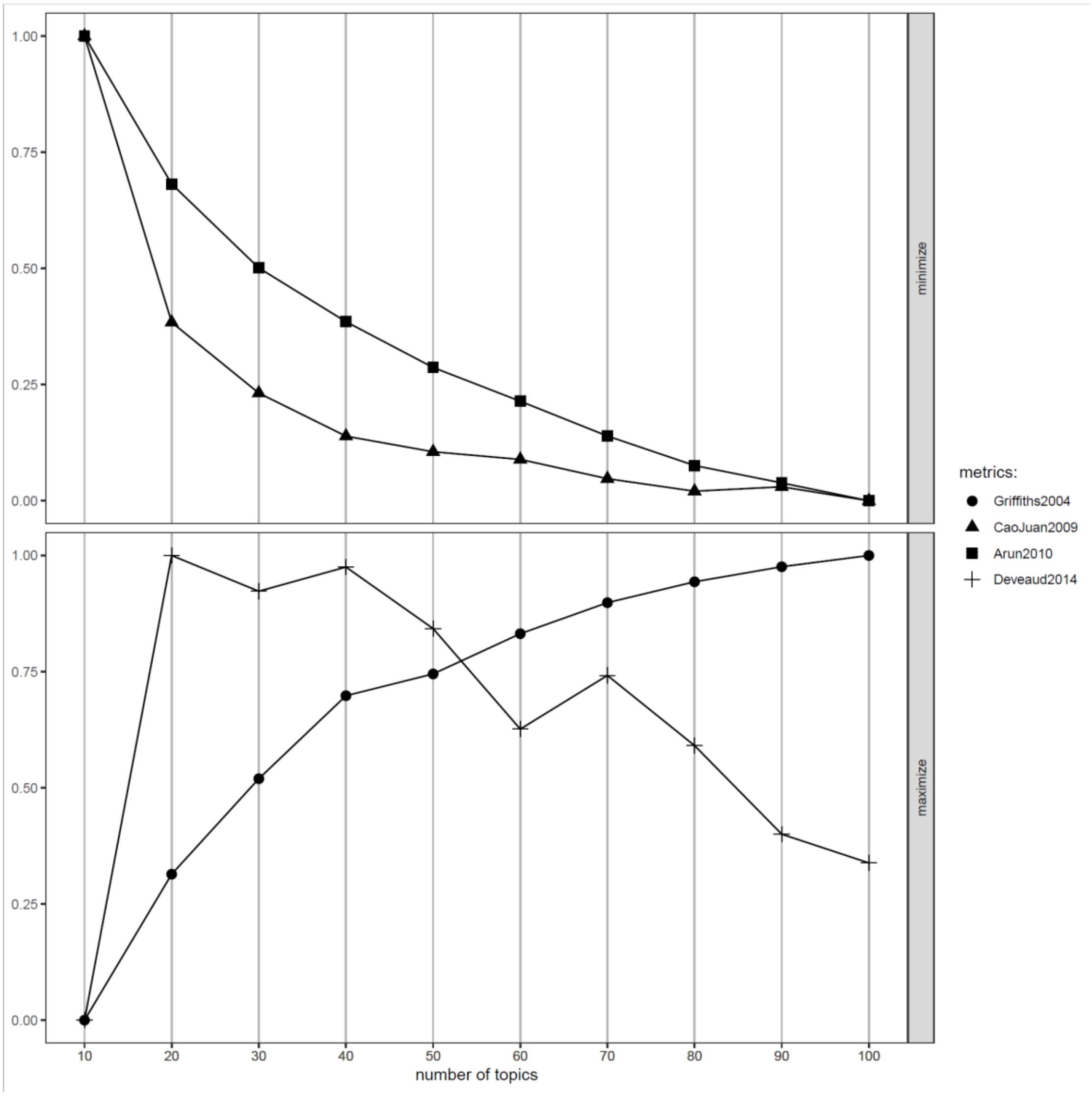

Ref:

1. Rajkumar Arun, V. Suresh, C. E. Veni Madhavan, and M. N. Narasimha Murthy. 2010. On finding the natural number of topics with latent dirichlet allocation: Some observations. In *Advances in knowledge discovery and data mining*, Mohammed J. Zaki, Jeffrey Xu Yu, Balaraman Ravindran and Vikram Pudi (eds.). Springer Berlin Heidelberg, 391–402. http://doi.org/10.1007/978-3-642-13657-3_43

2. Cao Juan, Xia Tian, Li Jintao, Zhang Yongdong, and Tang Sheng. 2009. A density-based method for adaptive lda model selection. *Neurocomputing — 16th European Symposium on Artificial Neural Networks 2008* 72, 7–9: 1775–1781. http://doi.org/10.1016/j.neucom.2008.06.011

3. Romain Deveaud, Éric SanJuan, and Patrice Bellot. 2014. Accurate and effective latent concept modeling for ad hoc information retrieval. *Document numérique* 17, 1: 61–84. http://doi.org/10.3166/dn.17.1.61-84
4. Thomas L. Griffiths and Mark Steyvers. 2004. Finding scientific topics. *Proceedings of the National Academy of Sciences* 101, suppl 1: 5228–5235. http://doi.org/10.1073/pnas.0307752101
5. Martin Ponweiser. 2012. Latent dirichlet allocation in r. Retrieved from http://epub.wu.ac.at/id/eprint/3558
